# Intrinsic connectomes underlying response to trauma-focused psychotherapy in post-traumatic stress disorder

**DOI:** 10.1038/s41398-020-00938-8

**Published:** 2020-08-05

**Authors:** Mayuresh S. Korgaonkar, Cassandra Chakouch, Isabella A. Breukelaar, May Erlinger, Kim L. Felmingham, David Forbes, Leanne M. Williams, Richard A. Bryant

**Affiliations:** 1 Brain Dynamics Centre, Westmead Institute for Medical Research, The University of Sydney, Sydney, NSW Australia; 2 Department of Psychiatry, Faculty of Medicine and Health, University of Sydney, Sydney, NSW Australia; 3 School of Psychology, University of New South Wales, Sydney, NSW Australia; 4 School of Psychological Sciences, University of Melbourne, Melbourne, VIC Australia; 5 Centenary of ANZAC Centre, a Department of Veterans’ Affairs funded initiative of Phoenix Australia, Carlton, VIC Australia; 6 Department of Psychiatry and Behavioral Sciences, Stanford University, Stanford, CA USA; 7 Sierra-Pacific Mental Illness Research, Education, and Clinical Center (MIRECC) VA Palo Alto Health Care System, Palo Alto, CA USA

**Keywords:** Prognostic markers, Psychiatric disorders

## Abstract

Although trauma-focused cognitive behavior therapy (TF-CBT) is the frontline treatment for post-traumatic stress disorder (PTSD), up to one-half of patients are treatment nonresponders. To understand treatment nonresponse, it is important to understand the neural mechanisms of TF-CBT. Here, we used whole-brain intrinsic functional connectivity analysis to identify neural connectomic signatures of treatment outcome. In total, 36 PTSD patients and 36 healthy individuals underwent functional MRI at pre-treatment baseline. Patients then underwent nine sessions of TF-CBT and completed clinical and follow-up MRIs. We used an established large-scale brain network atlas to parcellate the brain into 343 brain regions. Pairwise intrinsic task-free functional connectivity was calculated and used to identify pre-treatment connectomic features that were correlated with reduction of PTSD severity from pretreatment to post treatment. We formed a composite metric of intrinsic connections associated with therapeutic outcome, and then interrogated this composite metric to determine if it distinguished PTSD treatment responders and nonresponders from healthy control status and changed post treatment. Lower pre-treatment connectivity for the cingulo-opercular, salience, default mode, dorsal attention, and frontoparietal executive control brain networks was associated with treatment improvement. Treatment responders had lower while nonresponders had significantly greater connectivity than controls at pretreatment. With therapy, connectivity significantly increased for responders and decreased for nonresponders, while controls remain unchanged over this time period. We provide evidence that the intrinsic functional architecture of the brain, specifically connectivity within and between brain networks associated with external vigilance, self-awareness, and cognitive control, may characterize a positive response to TF-CBT for PTSD.

## Introduction

Trauma-focused cognitive behavior therapy (TF-CBT) is the preferred treatment for post-traumatic stress disorder (PTSD), yet at least half of patients do not respond to this approach^1,2^. To understand why only some people respond to treatment, it is crucial to elucidate the neurobiological mechanisms of TF-CBT. Functional magnetic resonance imaging (fMRI) studies have shed light on some of the neurobiological predictors of this treatment, with the predictive patterns of activations in specific regions, depending on the task employed^3–5^. A review of these studies concluded that TF-CBT response was predicted by elevated dorsal anterior cingulate prior to treatment, whereas elevated amygdala and insula activation was associated with treatment failure^6^. In terms of the effects of TF-CBT, other studies have found that TF-CBT resulted in decreased activation of the amygdala and insula, and increased activation in the dorsal anterior cingulate cortex and hippocampus^6^. Limited studies have also examined how task- dependent connectivity associated with some of these brain regions correlates with treatment, with greater suppression of amygdala–insula connectivity during cognitive reappraisal of negative-valence images^7^, and that of insula–pregenual anterior cingulate connectivity during processing of sad facial expressions associated with symptom improvement^8^.

While such a focused approach has been useful to identify neural regions underlying TF-CBT response, it is limited because it does not address how treatment response may be predicted by task-independent core-intrinsic connectivity within and between brain networks. The few studies investigating changes in resting-state connectivity following TF-CBT have reported increases between the amygdala and hippocampal limbic network structures with the executive top-down control prefrontal brain regions^9^, and a reduction in salience network connectivity^10^. Two recent studies have also evaluated the extent to which pre-treatment resting connectivity is associated with TF-CBT response. The first study used a combination of resting-state connectivity within the ventral attention network and delayed recall performance in a verbal memory task to predict the response to TF-CBT^11^. The second study identified that pre-treatment superior frontal and presupplementary motor-resting connectivity could distinguish veterans with PTSD who respond to TF-CBT from nonresponders^12^. However, these studies are also limited in that they focus either on specific regions or networks, and lack detailed examination of all connections in the brain.

Mapping the functional architecture of the entire brain, commonly referred to as the functional connectome, allows a holistic and integrated system-level understanding of how intrinsic functional networks of the brain are associated with treatment response. This approach can offer novel insights into how existing treatments work, to develop new treatments and potentially lead to new biomarkers for monitoring the effects of treatment. PTSD has been characterized by disruptions within these intrinsic brain networks^13–15^. For example, enhanced connectivity of brain regions associated with salience processing and hypervigilance (i.e., the salience network) but at the expense of awareness of internally focused thoughts, autobiographical memory, and compromised cognitive abilities (i.e., weakly connected and hypoactive default mode (DMN) and frontoparietal (FPN) or central executive brain networks, respectively) underlies PTSD^16^.

Despite the number of studies investigating the relationship between task-driven activation and connectivity of specific neural regions and treatment response to PTSD^6,17^, no prior studies have reported a systematic connectome-wide inspection of the detailed intrinsic functional architecture of the entire brain in the context of TF-CBT. Here, we adopted a comprehensive, connectome-wide approach^18^ to investigate large-scale intrinsic functional brain networks prior to treatment that characterize response to TF-CBT. We analyzed intrinsic functional connectivity from fMRI scans collected prior to and following a 9-week course of TF-CBT. We also examined whether connectivity within the response-related brain networks at baseline differs between PTSD and healthy participants, and whether it changes with treatment. We hypothesized that pre-treatment functional connectivity related to the salience, limbic, and frontoparietal brain networks is likely to characterize response to TF-CBT, to differ between PTSD and healthy individuals, and that connectivity will change following treatment.

## Materials and methods

### Participants and study protocol

Participant recruitment for the study commenced from August 2009. Participants were 51 treatment-seeking patients, 36 of whom had viable imaging data at baseline and 25 of these with follow-up MRI data (mean age 39.7 ± 11.3 years, 17 females; see CONSORT diagram in Supplementary Fig. 1 and participant characteristics in Table 1); PTSD, as defined by DSM-IV, was diagnosed by Masters-level clinical psychologists using the Clinician Administered PTSD Scale (CAPS^19^). Participants with a history of neurological disorder, moderate or severe traumatic brain injury, psychosis, bipolar disorder, or substance dependence were excluded. The protocol permitted prescribed medication if the dosage had remained stable for 2 months prior to the scan, and was not altered during the course of the study; 10 (27.8%) participants were taking selective serotonin-reuptake inhibitors.

**Table 1:** Participant characteristics for PTSD (treatment responders and nonresponders) and healthy controls.

	PTSD (*n* = 36)	Controls (*n* = 36)	Treatment responders (*n* = 25)	Treatment nonresponders (*n* = 11)
Age, mean (SD)	39.7 ± 11.3	38.3 ± 12.9	40.2 ± 12.4	38.6 ± 8.6
Male, *n* (%)	53%	50%	48%	63.6%
Time since trauma, months mean (SD)	17.5 ± 14.0	—	18.6 ± 15.3	14.9 ± 10.6
Type of trauma, *n* (%)		—		
Childhood abuse	3 (8.3)	—	3 (12)	0 (0)
Motor vehicle accident	5 (13.9)	—	2 (8)	3 (27.3)
Police-related trauma	10 (27.8)	—	8 (32)	3 (27.3)
Assault	14 (38.9)	—	9 (36)	5 (36.4)
Witness	3 (8.3)	—	3 (12)	0 (0)
Prescribed SSRI, *n* (%)	10 (27.8)	—	8 (32)	2 (18.2)
Major depressive disorder, *n* (%)	19 (52.8)	—	12 (48)	7 (63.6)
Social phobia, *n* (%)	15 (41.7)	—	8 (32)	7 (63.6)
Panic disorder, *n* (%)	5 (13.9)	—	4 (16)	1 (9.1)
Agoraphobia, *n* (%)	23 (63.9)	—	17 (68)	6 (54.6)
Obsessive compulsive disorder, *n* (%)	4 (11.1)	—	2 (8)	2 (18.2)
Generalized anxiety disorder, *n* (%)	12 (33.3)	—	8 (32)	4 (36.4)
DASS depression, mean (SD)	10.9 ± 5.5	—	10.5 ± 5.8	11.8 ± 3.9
DASS anxiety, mean (SD)	8.1 ± 4.4	—	7.2 ± 4.8	10.1 ± 2.3
DASS stress, mean (SD)	12.0 ± 4.4	—	11.4 ± 4.6	13.5 ± 3.5
Baseline CAPS severity, mean (SD)	72.2 ± 14.0	—	71.9 ± 20.1	72.7 ± 11.1
Post-treatment CAPS severity, mean (SD)	27.5 ± 19.6	—	17.2 ± 12.1*	51.0 ± 10.8*

*PTSD* post-traumatic stress disorder, *CAPS* clinician-administered PTSD scale, *DASS21* depression anxiety stress scale—21 items.

* indicates a significant difference at *P* < 0.05 between treatment responders and nonresponders.

The study also included a comparison group of 36 healthy participants (mean age 38.3 ± 12.9 years, 18 females) screened using the Mini International Neuropsychiatric Interview (MINI version 5.5^20^). All controls underwent baseline MRI scans, and 22/36 controls completed follow-up scans. Depression and anxiety levels were also assessed by self-report on the depression, anxiety, and stress scale (DASS^21^).

The authors assert that all procedures contributing to this work comply with the ethical standards of the relevant national and institutional committees on human experimentation, and with the Helsinki Declaration of 1975, as revised in 2008. The study was approved by the Western Sydney Area Health Service Human Research Ethics Committee (Approval#ETH00309), and written informed consent was obtained from participants.

### Treatment protocol

Within 2–3 weeks of scanning, participants commenced a course of nine once-weekly individual sessions of TF-CBT that were delivered by experienced doctoral-level or masters-level clinical psychologists. This therapy involved an initial session of psychoeducation about psychological responses to trauma, then six sessions of 40-min imaginal exposure to the trauma memory, instructions regarding in vivo exposure to avoided situations, and cognitive restructuring of thoughts related to the traumatic event^22^. An additional session reinforced cognitive restructuring exercises, and a final session focused on relapse prevention. This therapy procedure is consistent with gold-standard TF-CBT protocols^23^. Independent clinicians rated the fidelity of 130 sessions (18%), indicating full adherence to the treatment protocols and high level of quality on a 7-point scale (mean ± SD = 6.11 ± 1.32). A post-treatment assessment using the CAPS was conducted by an independent clinical psychologist 1 week following completion of the course of treatment. To examine change in PTSD severity independent of initial severity, residual change was calculated from a regression of pre-treatment total CAPS scores on post-treatment total CAPS scores^24^ (higher change scores correspond to greater improvement), and was correlated with neural measures.

### fMRI acquisition, preprocessing, and generation of functional connectomes

Details of MRI acquisition, activation tasks, preprocessing, and intrinsic functional connectivity estimation were published previously^25–27^ and can be found in Supplementary Materials. In brief, MRI data for both visits were acquired on a 3 T GE Signa HDx scanner using an 8-channel head coil. MRI acquisition included five fMRI tasks (a Go/NoGo cognitive task, conscious and nonconscious emotion face processing, and two runs of cognitive reappraisal task) (echo-planar imaging; TR/TE = 2500/27.5 ms, flip angle = 90°, 64 × 64 matrix, 40 axial 3.5-mm slices, and 120 volumes) and a 3D T1-weighted structural MRI scan (TR/TE = 8.3/3.2 ms, flip angle = 11°, TI = 500 ms, 256 × 256 matrix, and 180 sagittal 1-mm slices). Intrinsic functional connectivity was estimated using data from all five tasks. fMRI images were motion-corrected and corrected for geometric distortions using realignment and unwarping, slice time corrected, spatially normalized to the stereotactic MNI space, and smoothed. As motion is a critical issue in resting-state data, data volumes associated with high movement (framewise displacement from one time point to the next) or changes in BOLD signal intensity were censored (temporally masked) to reduce the influence of motion and related artifacts^28,29^. For each fMRI task, the BOLD responses for each experimental condition were modeled in the general linear model framework along with the mean signal time course extracted from eroded ventricle and white matter masks, as well as the temporal masks derived from the volume censoring described above, and motion effects using the Volterra expansion of the realignment parameters. To isolate an estimate of intrinsic functional connectivity, we regressed voxelwise BOLD time series against the model incorporating task covariates as nuisance signals and analyzed the residuals of this model. Subsequent to this denoising procedure, the time series were band-pass filtered (0.009 Hz < f < 0.08 Hz). Intrinsic connectivity estimated using this approach has been previously validated with task-free resting-state connectivity^30^.

Functional connectomes were generated and analyzed using our previously published procedure^27^. We parcellated every individual’s brain image into 343 brain regions or nodes using a high-resolution 333 cortical template based on Gordon et al.^31^ and 10 subcortical regions obtained from the AAL atlas. This template uses resting-state functional connectivity patterns to define brain parcels that represent putative, functionally coherent, brain areas providing a label based on intrinsic functional brain networks. Intrinsic functional time series were extracted for each of the regions and correlated with every other region to obtain a 343 × 343 interregional functional connectivity matrix for every individual. We transformed the correlation coefficients into *z* scores using Fisher’s *z* transformation. The specific choice of a parcellation scheme can impact the results of a network analysis^32–34^; hence, we tested the robustness of findings using a second anatomical parcellation based on the AAL atlas^35^ (see Supplementary Materials).

### Statistical analyses

The statistical analysis was designed in a stepwise manner to address the study aims as follows.

#### To identify connectome features associated with TF-CBT treatment response

The network-based statistic (NBS)^36^ was used to assess associations between pre-treatment functional connectivity and improvement in PTSD symptoms. Analogous to cluster-based correction strategies used in voxelwise MRI studies, the NBS deals with the multiple-comparison problem posed by connectomic data by evaluating the null hypothesis at the level of interconnected subnetworks rather than individual connections. We used a primary component- forming threshold of *P* < 0.001 to identify all significant interregional connections correlated with symptom improvement, and then tested for statistical significance of the size of the connected components relative to an empirical null distribution generated by random shuffling of the order of individuals within the group using 5000 permutations. This gives a corrected *P* value for each observed component.

Observed components with *P* < 0.05, componentwise corrected, were identified as significant subnetworks associated with treatment improvement. Functional connectivity estimates for each interregional connection of the identified subnetwork were extracted. We computed a single composite connectivity metric averaged across all the significant connections of this subnetwork for the subsequent analyses below. To unpack findings further at the known intrinsic network level, we computed an average connectivity estimate for each of the labeled intrinsic functional network pair combinations that characterized this network (see Supplementary Table S1). To control for multiple testing due to the number of measures, we employed a Benjamini–Hochberg FDR-corrected *P* < 0.05 for statistical evaluation.

In supplementary analyses, we tested for associations between connectivity with demographic and clinical symptom measures, and retested associations with treatment improvement controlling for these measures. To understand the individual-level predictive value and provide an operational example of how the connectivity markers we identified could be helpful in a treatment decision, and to inform future studies, we tested the average connectivity estimates for the network as potential predictors of treatment outcome (quantified as a binary response variable corresponding to a 50% decrease in symptoms) using leave-one-out cross-validation analyses. We evaluate the additive predictive value relative to demographic and clinical measures in this analysis.

#### Does the treatment response-related connectomic feature also characterize PTSD disease state at baseline?

We compared the composite connectivity metrics (i.e., average for the identified network feature and also average connectivity estimates for each labeled intrinsic functional network pair combinations that characterized this network feature) using an ANOVA with group (PTSD/control) as a between-participant factor. In addition, we also split the PTSD group into responders and nonresponders (with responders defined as having at least 50% improvement in symptoms) and evaluated each group separately relative to controls. This was done to evaluate if responders or nonresponders have a similar/abnormal connectivity profile relative to healthy individuals prior to treatment. To investigate beyond the identified subnetwork, we also performed an exploratory whole-brain connectivity comparison between the PTSD and control groups using NBS (Supplementary Analyses).

#### Does the treatment response-related connectomic feature change after treatment?

We used an ANOVA with pre- versus post assessment (time) as a within-participant factor and group (with levels for PTSD depending on treatment response and controls) as a between-participant factor to evaluate treatment effects on changes in the composite connectivity metrics described above. We tested for the interaction between pre–post assessment and group, and performed post hoc tests to characterize any significant interaction.

For all analyses above, we controlled multiple testing using the Benjamini–Hochberg FDR- corrected *P* < 0.05 for statistical evaluation.

## Results

### Pre-treatment connectome features associated with TF-CBT treatment response

**Fig. 1:**
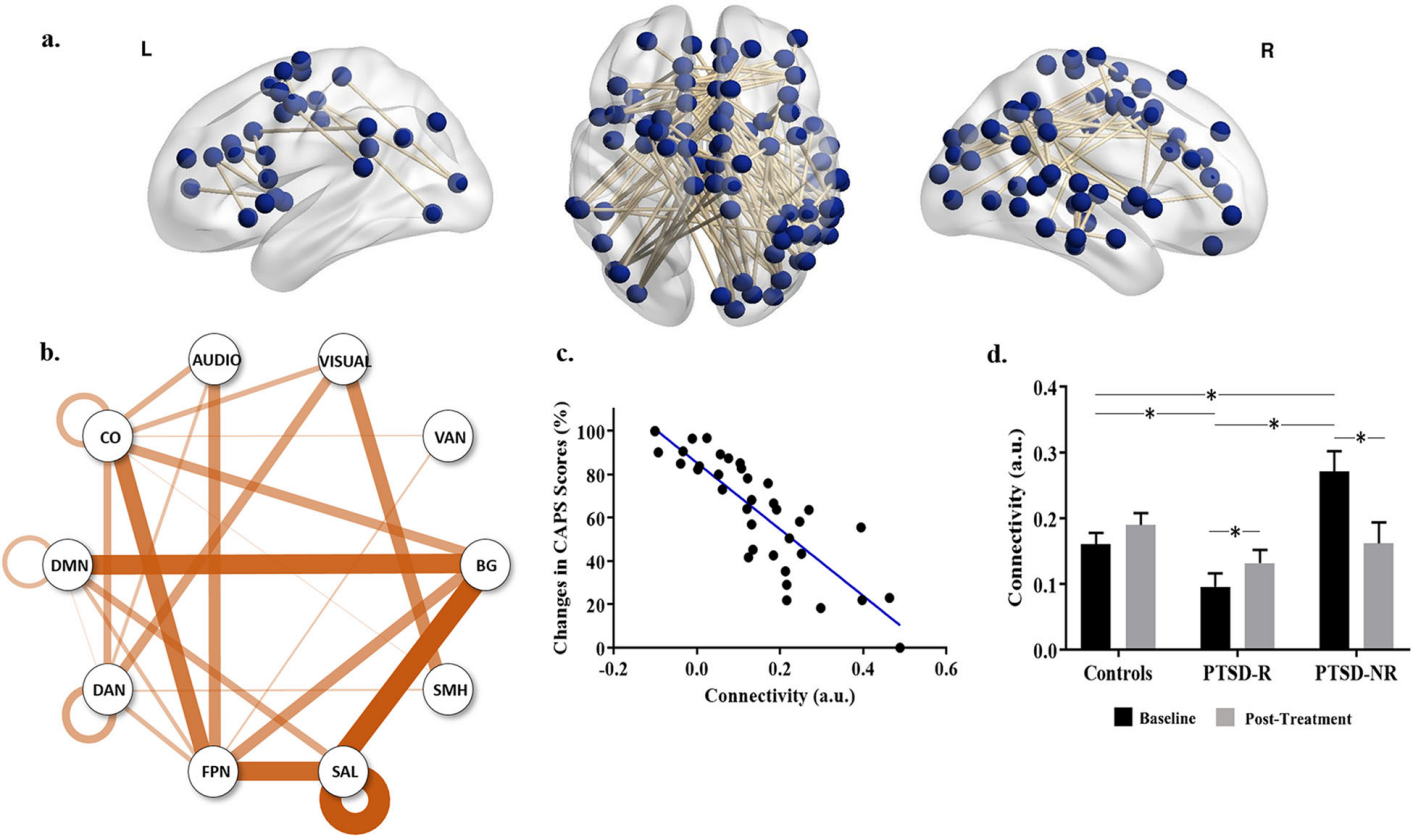
Pre-treatment functional connectivity across the whole-brain network in PTSD patients associated with symptom improvement following TF-CBT. The connectomic feature identified from the NBS analysis is shown below (**a**) from sagittal and axial views. (**b**) Intra- (loops) and internetwork connections characterizing this connectomic feature are shown. The thickness of the line corresponds to the number of significant connections relative to all possible connections between two networks, i.e., thicker lines imply more number of connections between networks. **c** Hypoconnectivity in this network at baseline corresponds with greater changes in CAPS scores and subsequently greater improvement in symptoms, such that (**d**) connectivity in this network at baseline is distinct between responders, nonresponders, and controls. Connectivity within the PTSD-response groups is normalized post treatment. Bar plots (means and SD) showing average connectivity estimates at baseline and post treatment for each group. Asterisks indicate significant post hoc findings (*P* < 0.05) for this interaction. BG basal ganglia, CO cingulo-opercular, DAN dorsal attention, DMN default-mode network, FPN frontoparietal, SAL salience, SMH somatomotor hand, VAN ventral attention.

The NBS analysis identified a connectomic signature comprising 122 interregional connections between 88 brain nodes, which was significantly associated with treatment response (lower baseline connectivity associated with greater improvement in symptoms; *P* = 0.044 corrected for multiple comparisons, Fig. 1).

This connectomic signature was largely characterized by (a) lower intranetwork intrinsic functional connectivity associated with treatment improvement within the cingulo-opercular, DMN, dorsal attention, and salience networks; also, lower internetwork connectivity associated with treatment improvement; (b) between the regions of the cingulo-opercular, DMN, dorsal attention, and frontoparietal networks; (c) between regions of the cingulo-opercular, dorsal attention, and frontoparietal networks with auditory and visual networks; (d) between basal ganglia regions of the subcortical network with the DMN, cingulo-opercular, frontoparietal, and salience networks; (e) between the visual and somatomotor networks. Connectivity was associated with anxiety levels measured on the DASS and the presence of a comorbid condition, but remained significantly associated with improvement in symptoms controlling for both these measures.

This connectivity signature significantly improved predictive accuracy in classifying responders from nonresponders compared with a model using demographic and clinical measures alone (*P* < 0.001; Supplementary Table S2: cross-validated accuracy of the model without connectivity/with connectivity = 64.3%/71.4%, sensitivity = 25.0%/100.0%, and specificity = 80.0%/60.0%). Finally, the connectivity signature was replicated using the second parcellation scheme (Supplementary Table S3).

### Does the treatment response-related connectomic feature also characterize PTSD at baseline?

At baseline, controls were not significantly different compared with the whole PTSD cohort for connectivity in this signature (*P* > 0.05). However, responders had a significantly lower average connectivity than controls (*P* = 0.021); in contrast, nonresponders had a significantly greater average connectivity than controls (*P* = 0.017). Specifically, only connections between the frontoparietal and cingulo-opercular brain networks were found significant after correcting for multiple comparisons where nonresponders had significantly greater connectivity than controls (FDR *P* < 0.001, Table 2).

**Table 2:** Functional connectivity across the significant whole-brain network at baseline and following TF-CBT treatment.

Networks	Functional connectivity (mean ± SD)						*P* values									
	Ctrl		PTSD-R		PTSD-NR		Time*group	Main effect time (baseline vs. post)			Baseline only—between groups			Post Tx only— between group		
	Baseline	Post	Baseline	Post	Baseline	Post		PSTD-R	PTSD-NR	Ctrl	PTSD-R < PTSD-NR	PTSD-NR > Ctrl	PTSD-R < Ctrl	PTSD-R < PTSD-NR	PTSD-NR > Ctrl	PTSD-R < Ctrl
Whole network	0.161 ± 0.017	0.190 ± 0.018	0.096 ± 0.020	0.132 ± 0.020	0.271 ± 0.031	0.162 ± 0.032	<0.000*	0.011	0.015	NS	*<0.001	0.017	0.021	NS	NS	NS
Auditory—cingulo-opercular	0.215 ± 0.029	0.246 ± 0.044	0.079 ± 0.035	0.041 ± 0.049	0.280 ± 0.053	0.087 ± 0.078	0.025	NS	0.021	NS	*0.002	NS	0.005	NS	NS	*0.003
Frontoparietal—cingulo-opercular	0.083 ± 0.024	0.161 ± 0.032	0.085 ± 0.029	0.123 ± 0.035	0.301 ± 0.044	0.151 ± 0.057	0.012	NS	NS	0.045	*<0.001	*<0.001	NS	NS	NS	NS
Frontoparietal—DMN	0.137 ± 0.032	0.211 ± 0.050	0.046 ± 0.038	0.150 ± 0.056	0.276 ± 0.058	0.178 ± 0.089	0.009	0.02	NS	NS	*0.004	0.032	NS	NS	NS	NS
Frontopariet—ventral attention	0.126 ± 0.036	0.137 ± 0.042	−0.025 ± 0.043	0.033 ± 0.046	0.149 ± 0.065	0.007 ± 0.074	0.027	NS	NS	NS	NS	NS	0.004	NS	NS	NS
Dorsal attention—visual	0.133 ± 0.023	0.135 ± 0.036	0.060 ± .028	0.076 ± 0.040	0.235 ± 0.042	0.083 ± 0.064	0.017	NS	0.032	NS	*0.002	0.034	NS	NS	NS	N
DMN—subcortical	0.158 ± 0.024	0.138 ± 0.029	0.087 ± 0.029	0.137 ± 0.032	0.241 ± 0.044	0.141 ± 0.051	0.022	NS	NS	NS	*0.007	NS	NS	NS	NS	NS
Somatomotor (hand)—visual	0.325 ± 0.160	0.139 ± 0.032	0.020 ± 0.193	0.114 ± 0.036	0.159 ± 0.290	0.053 ± 0.057	0.001*	0.001	NS	NS	*0.002	NS	NS	NS	NS	NS

Network links with a significant time*group interaction are listed (*significant at FDR-corrected *P* < 0.05).

### Does the treatment response-related connectomic feature change after treatment?

**Fig. 2:**
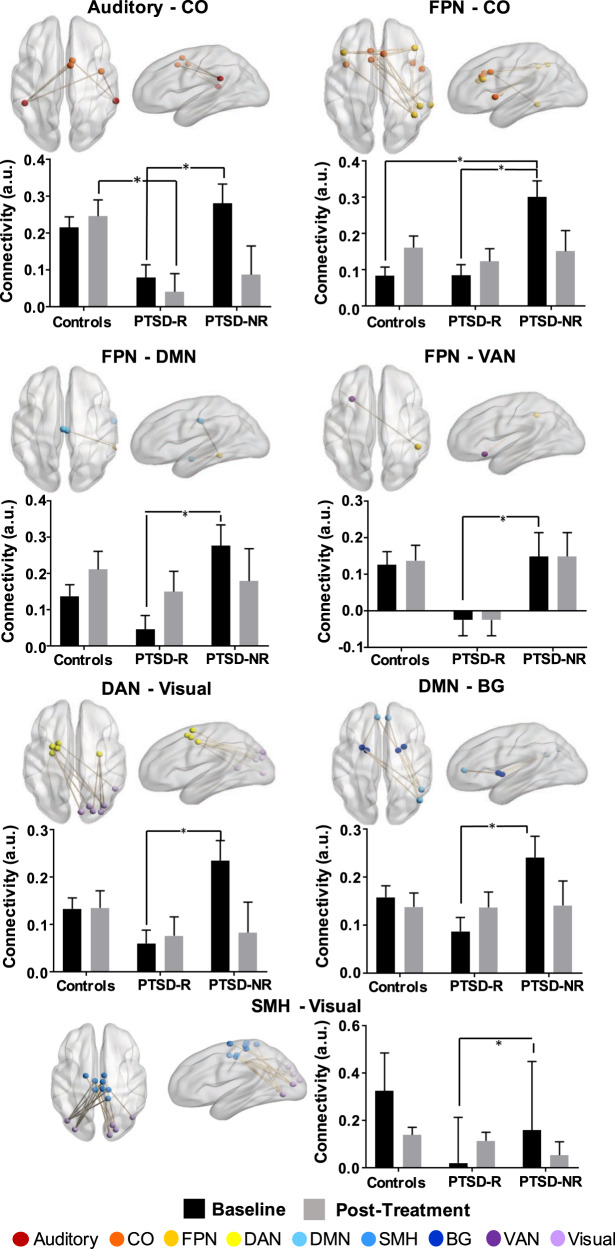
Pre- and post-treatment functional connectivity across the significant networks that were associated with PTSD symptom improvement following TF-CBT. Network links with a significant Time*Group interaction are shown. This includes connectivity between the (**a**) auditory and CO network, (**b**) the FPN and CO network, (**c**) the FPN and DMN, (**d**) the FPN and VAN, (**e**) the DAN and visual network, (**f**) the DMN and regions of the basal ganglia, and (**e**) the SMH and visual network. *indicates post hoc comparisons significant at FDR *P* < 0.05. BG basal ganglia, CO cingulo-opercular, DMN default-mode network, DAN dorsal attention, FPN frontoparietal, SMH somatomotor hand, VAN ventral attention.

A significant group × time interaction was observed for average connectivity in this signature (*P* < 0.001), and specifically only for connections between the somatomotor and visual networks (FDR *P* < 0.05, Table 2 and Fig. 2). Post hoc contrasts indicated that average connectivity for this signature remained unchanged for controls over time, but increased for treatment responders (*P* = 0.011) and decreased for nonresponders (*P* = 0.015) following treatment. Connectivity for both responders and nonresponders was not different from controls post treatment. Only an increase in connectivity between visual and somatomotor networks was found significant in responders (FDR *P* = 0.001).

## Discussion

Using a comprehensive, connectome-wide analysis, we examined pre-treatment intrinsic functional connectivity associated with response to 9 weeks of TF-CBT treatment in PTSD individuals. To our knowledge, this is the first evidence of a connectome-wide inspection of intrinsic neural connectivity within and between key large-scale brain networks to predict response to TF-CBT in PTSD. We found an overall lower-than-normal pre-treatment connectivity to be associated with better treatment response, whereas greater-than-normal connectivity was associated with nonresponse to TF-CBT.

Previous studies have shown that brain regions in the limbic, cognitive, and salience brain networks were probed by functional tasks to be associated with TF-CBT^37^. Connectivity in these three networks has consistently been reported abnormal in PTSD^14^. Our study provides new evidence that task-free intrinsic functional connectivity in some of these brain networks is also associated with response to TF-CBT. This highlights that the core functional architecture of these networks is associated with treatment response. Specifically, we found lower connectivity in networks underlying vigilance and tonic alertness, such as cingulo-opercular, salience, and dorsal attention, to be associated with better response to TF-CBT. PTSD is characterized by hypervigilance to threat cues, which is reflected in hyperactivity and hyperconnectivity of brain regions associated with these networks during processing of threat stimuli^38^. It is possible that lower intrinsic connectivity in these networks prior to treatment reflects patients’ tendencies to not engage in excessive vigilance and reactivity, which may promote better response to behavioral strategies that involve reduced reactivity to threat. The basal ganglia, a group of subcortical nuclei, are also involved in voluntary component of the behavioral expression of emotion via their interaction with some of the threat-processing limbic brain regions such as the amygdala^39^. This may also explain the lower connectivity between the basal ganglia with the DMN, cingulo-opercular, frontoparietal, and salience networks observed to be associated with better treatment response.

We also observed that lower connectivity of the cingulo-opercular, salience, and dorsal attention networks with the frontoparietal cognitive control brain network was associated with better response. One of the main tenets for TF-CBT is the capacity to reappraise events and responses associated with the traumatic experience, such that they are experienced with less anxiety^40^. The cognitive control network of the brain is known to play a crucial role in reappraising traumatic events and experiences^41^. Moreover, impairments in this network have been shown to predict poor response to TF-CBT^42,43^. Our results seem to suggest that hypervigilance or greater tonic alertness, reflected as greater connectivity in cingulo-opercular, salience, and dorsal attention networks associated with these processes, could possibly be dampening reappraisal-related brain networks, thereby reducing the individual’s ability to utilize TF-CBT. Specifically, nonresponders in our analysis had abnormally higher connectivity between cingulo-opercular and frontoparietal brain networks. In contrast, PTSD individuals who have reduced connectivity between these networks, have better capacity to learn skills to reappraise traumatic experiences and hence respond to treatment due to a less-disturbing influence of this salience-related connectivity on the cognitive brain networks.

We also observed reduced connectivity within regions of the DMN, and between the DMN and the frontoparietal network, to be associated with better response to TF-CBT. The DMN is associated with self-referential processing and rumination of autobiographical memories, and is anti-correlated with networks involved in attending to functions such as attentional vigilance, cognitive control, and orienting^44^. A decreased DMN connectivity due to a shift in focus from internal thoughts to external vigilance has been observed in PTSD^45,46^. It is possible that reduced DMN-related connectivity associated with treatment improvement reflects a reduced focus on trauma memories, which leads to better treatment response. It is also possible that reduced coupling of the DMN with the frontoparietal brain regions could mean less interference with goal-oriented tasks^47^ such as learning reappraisal skills, and thereby benefit from TF-CBT.

PTSD is known to impact sensory processing, particularly visual processing. For example, severe emotional trauma produces recurrent and vivid unpleasant sensory recollections that impact visual processing even at the preattentive level^48^. In our analysis, lower connectivity related to the visual brain regions, particularly to the other sensory somatomotor brain regions, was associated with better treatment outcome with responders demonstrating an increase in this connectivity to normal levels following treatment.

To our knowledge, only two other studies have previously reported on pre-treatment resting fMRI connectivity to predict clinical outcome to TF-CBT in PTSD. Etkin et al.^11^ found that resting-state connectivity within the ventral attention network alone was not able to moderate treatment outcome. However, this connectivity in combination with a measure of verbal memory- delayed recall was able to predict treatment outcome with 85% accuracy. The other study by Zhutovsky et al.^12^ found an individual-level accuracy of 81.4% using resting-state connectivity associated with the presupplementary motor area to characterize responders. We observed a 71.4% accuracy in our analysis. Although we employed a cross-validation approach, these estimates should be interpreted with caution, considering the small-sample size and within- cohort cross-validation. Importantly, connectivity measures significantly improved the predictive capacity relative to demographic and clinical measures alone.

Our study also provides insight into how TF-CBT impacts neural connectivity based on heterogeneity of treatment response. TF-CBT has shown to increase amygdala and hippocampal connectivity to the orbitofrontal and medial prefrontal brain cortices^9^. This increase has been associated with improved capacity for inhibition and re-evaluation of threat and heightened memory ability^9^. Importantly, this increase in connectivity was to normal levels at the end of treatment. We did not specifically evaluate connectivity related to the amygdala and hippocampus because pre-treatment connectivity related to these regions was not associated with treatment outcome in our analysis. However, we observed that the effect of TF-CBT was to normalize intrinsic connectivity in our identified connectome feature in different ways for responders and nonresponders. PTSD patients who responded to TF-CBT had lower average connectivity in the identified connectomic feature prior to treatment, and demonstrated an increase in this connectivity (particularly in the somatomotor–visual and frontoparietal–DMN connections) to normal healthy levels following treatment; nonresponsive patients had greater connectivity at baseline (particularly in the dorsal attention–visual and cingulo-opercular–auditory/insula connections), which was also found to normalize post treatment. This might suggest that the overall effect of TF-CBT is to normalize the response-related intrinsic neural connections, irrespective of treatment outcome. These connectivity results complement findings from task-based activation studies that also observe differential changes of pre-treatment levels of recruitment in the salience (anterior cingulate and insula) and limbic (amygdala) brain regions following treatment^49–51^. These studies however lacked comparison with controls post treatment.

We note that previous studies have reported that resting-state connectivity is altered in PTSD^14^. However, we did not observe connectivity differences between the PTSD cohort as a whole and controls either at the whole-brain connectome level or within the network-identified feature associated with treatment response. Noting that the majority of previous work has focused on specific networks or resting-state connectivity related to specific regions of interest^14^, it is likely that our available sample size is limited to have enough power to identify whole-brain connectome differences relative to controls. It is also likely that the specific identified prognostic network feature may not be able to characterize PTSD diagnosis.

The following limitations should be considered. We did not include a wait-list comparison group, which would have provided a stricter index of the predictive capacity of baseline neural measures on TF-CBT relative to spontaneous remission. However, spontaneous remission is unlikely in our sample for which the mean time since trauma exposure was 3 years, and most spontaneous remission from PTSD occurs within the first 12 months. Our sample size is relatively small for building predictive models, and these models are likely prone to overfitting. Also, although we employed a leave-one-out cross-validation, we are testing a feature in the same sample that was used to identify that feature, this could overestimate the generalizability. Our overall goal with this analysis was primarily to evaluate if the connectivity measures contribute significantly on top of easily obtainable demographic and clinical measures, and importantly understand the neural mechanisms of treatment response rather than evaluate predictive utility. Testing this predictive model in a larger and independent cohort would give a more reliable estimate of the predictive capacity of the identified neural features. Some of the PTSD patients in our study also failed to complete the imaging protocol at follow-up, which reduced the available sample size for the follow-up imaging data, especially after splitting responders and nonresponders. Hence, our follow-up findings should be interpreted with caution, and need to be tested using a large cohort. A proportion of participants were using SSRIs. However, supplementary analyses indicated that all significant results were observed in nonmedicated participants (Supplementary Table S4).

In summary, lower-than-normal intrinsic connectivity within brain networks associated with external vigilance, self-awareness, and cognitive control may be a key mechanism for optimal response to TF-CBT. The effect of TF-CBT is to normalize this network architecture. Functionally probed activation and connectivity related to these networks are known to underlie the response to TF-CBT in PTSD. Our results suggest that this relationship extends beyond the contextual task, and the core underlying functional architecture of these networks is equally important in understanding the response to TF-CBT. This provides new insight into understanding the neural circuit mechanisms of TF-CBT in PTSD.

## Supplementary information

Supplementary Information
