# Supplementary material for: Intrinsic connectomes underlying response to trauma-focused psychotherapy in post-traumatic stress disorder
Source: Transl Psychiatry. 2020 Aug 5;10:270. doi: 10.1038/s41398-020-00938-8 (PMC7406502; doi:10.1038/s41398-020-00938-8)
Supplement: Supplementary file 1 — Supplementary Information [file 41398_2020_938_MOESM1_ESM.docx]

**Intrinsic Connectomes Underlying Response to**

**Trauma-Focused Psychotherapy in Posttraumatic Stress Disorder**

*Running Title: Connectome signatures of CBT response*

Mayuresh S. Korgaonkar^1, 2^, Cassandra Chakouch^1^, Isabella A. Breukelaar^1, 3^, May Erlinger^1^, Kim L. Felmingham^4^, David Forbes^5^, Leanne M. Williams^6, 7^, and Richard A. Bryant^1, 3^

^1^Brain Dynamics Centre, Westmead Institute for Medical Research, The University of Sydney.

^2^Department of Psychiatry, Faculty of Medicine and Health University of Sydney

^3^School of Psychology, University of New South Wales

^4^Discipline of Psychological Science, University of Melbourne

^5^Phoenix Australia Centre for Posttraumatic Mental Health, University of Melbourne

^6^Department of Psychiatry and Behavioral Sciences, Stanford University

^7^Sierra-Pacific Mental Illness Research, Education, and Clinical Center (MIRECC) VA Palo Alto Health Care System, Palo Alto

**Table of contents:**

S1: fMRI acquisition and preprocessing methods

S2: Associations of connectivity with demographic and clinical measures

S3: Predictive models using the identified connectomic signature in classifying responders from non-responders prior to treatment

S4: Network-Based Statistical analyses comparing whole PTSD group with Controls

S5: Replication of main connectome analyses using the AAL parcellation scheme

S6: Testing effects of current antidepressant medication use on findings

Supplementary Figure 1: CONSORT Diagram for the study

Supplementary Table S1: Network-based statistical analysis identifying the relationship between baseline functional connectivity and improvement in CAPS scores for PSTD patients

Supplementary Table S2: Predictive Models using demographic/clinical measures alone and combined models using connectivity measures for the identified connectomic signatures.

Supplementary Table S3: Pretreatment network identified to be significant with improvement in symptoms using network-based statistical analysis based on parcellations derived from the AAL atlas

Supplementary Table S4: Pretreatment functional connectivity associations with improvement in PTSD CAPS scores in non-medicated PTSD patient group


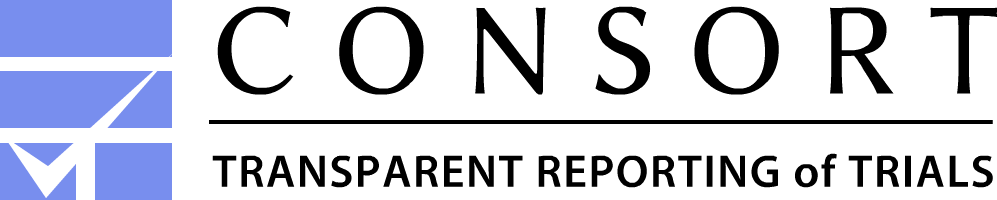


**Supplementary Figure S1: CONSORT 2010 Flow Diagram**

Analysed (n = 36 with n=25 for follow-up MRIs)
♦ Excluded from analysis because of MRI movement (n = 4)

Allocated to TF-CBT (n= 84)

♦ Consented to MRI assessment (n = 51)

## Follow-Up

## Analysis

Not included in MRI study

Not included in MRI study

Lost to follow-up (n = 11)

Did not complete follow-up MRIs (n=15)

## Enrollment

## Allocation

Allocated to Control conditions (n= 72)

♦ Consented to MRI assessment (n = 0)

Randomized (n= 156)

Excluded (n= 48)

♦  Not meeting inclusion criteria (n= 28 )

♦  Declined to participate (n=8 )

♦  Other reasons (n=20 )

Assessed for eligibility (n= 204)

**S1: fMRI acquisition and preprocessing methods**

MRI data for both visits was acquired on a 3T GE Signa HDx scanner (GE Healthcare, Wisconsin) using an 8-channel head coil. MRI acquisition included five fMRI tasks and a 3D T1-weighted structural MRI scan (TR/TE=8.3/3.2ms, Flip Angle=11°, TI=500ms, 256x256 matrix, 180 sagittal 1mm slices, 1mm isotropic voxels). MR images for each task were acquired using echo planar imaging (TR/TE=2500/27.5ms, Flip Angle=90°, 64x64 matrix, FOV=24cm, 40 axial 3.5mm slices covering the whole brain, 120 volumes). The details of the five tasks have been previously described (1, 2). Briefly, tasks comprised of (a) the Go-No-Go task that typically evaluates inhibition processing, (b) conscious and (c) non-conscious processing of emotional faces, and two iterations of an emotional cognitive reappraisal task with (d) instructions to either passively watch negative or neutral valence IAPS pictures (‘Watch’ emotional or neutral condition) or cognitively reappraise (‘Think’ condition) negative valence pictures in a block design. The order of the instructions i.e. condition blocks for this task were counterbalanced across participants.

To ensure that participants in the PTSD group were adequately controlled for age/gender, fMRI data from 9 of the 36 participants in the control group were sourced from a previous study. Instead of completing two iterations of the emotional regulation task these participants completed an auditory oddball task that evaluates selective attention and a continuous performance task examining working memory. There were no differences in connectivity estimates for these control individuals as compared to the remaining healthy individuals. For all participants, intrinsic functional connectivity was estimated using data from a combination of five tasks as described below.

***Preprocessing of Connectivity Data***

Data was preprocessed and analyzed using the Statistical Parametric Mapping software (SPM8; Wellcome Department of Cognitive Neurology, London) implemented in Matlab. First images were motion-corrected and corrected for geometric distortions using realignment and unwarping, and then quality control diagnostics were completed on the time series data for each run. To reduce the influence of motion and related artifacts, data volumes that were associated with extreme (i) movement (framewise displacement from one time point to the next) or (ii) changes in BOLD signal intensity (as indexed by spatial standard deviation of successive difference images [DVARS] (3) were censored (temporally masked). Framewise displacement was calculated as the sum of the absolute values of the differentiated realignment estimates as described in Power et al., 2014 (4). The criteria for censoring volumes was based on established thresholds of framewise displacement that exceeded or were equal to 0.3mm, and scaled signal intensity differences greater than 10 (3-5). Censoring was implemented using the time series difference analysis toolbox http://www.fil.ion.ucl.ac.uk/spm/ext/#TSDiffAna and in house scripts. A temporal mask was then created for each censored volume and used as regressors of no interest in the first level statistical model (3, 4). Since movement related artifacts have been shown to impact volumes acquired before and several seconds after a movement spike, to reduce the influence of movement related artifacts a total of four temporal masks were created for each movement spike (an additional volume before and 2 volumes after the movement spike) (4). For signal change spikes, the temporal mask included the censored volume as well as one subsequent volume. Images were then slice-time corrected in SPM8. Following slice time correction, images were spatially normalized to the stereotactic Montreal Neurological Institute (MNI) space using the FMRIB nonlinear registration tool (6) using the T1 structural data and smoothed using an 8 mm full-width-at-half-maximum Gaussian kernel in SPM8.

For each fMRI task, the BOLD responses for each experimental condition were modeled in the general linear model framework: Go-NoGo tasks (Go and NoGo trials), both emotion tasks (each emotion type) and both iterations of the emotion reappraisal task (Watch and Think trials).
For control participants that were sourced from a previous study the oddball (target and non-target trials) and continuous performance (working memory, 1-back and baseline trials) tasks were modeled in the general linear model framework in the absence of the emotion reappraisal task.
Motion effects were also modeled for each task using the Volterra expansion of the realignment parameters proposed in (7) (24 regressors; R_t_, R_t_^2^, R_t-1_, R_t-1_^2^ where R’s are the realignment parameters estimated during the preprocessing stage). Additional covariates for each task included the mean signal time course extracted from eroded ventricle and white matter masks, as well as the temporal masks derived from the volume censoring described above. To isolate an estimate of intrinsic functional connectivity, we regressed voxelwise BOLD time series against the model incorporating task covariates as nuisance signals and analysed the residuals of this model. Subsequent to this denoising procedure, the timeseries were band-pass filtered (0.009 Hz < f < 0.08 Hz). Intrinsic connectivity estimated using this approach has been previously validated with task-free resting state connectivity (8).

**S2: Associations of connectivity with demographic and clinical measures**

We performed uni- and multi-variate ANOVA analyses for each demographic and clinical measure to test associations with functional connectivity for the whole PTSD group. Univariate analyses were conducted to test associations with a single connectivity estimate averaged across the significant links for the identified network, whilst multivariate analyses tested the average connectivity estimates for the 25 intrinsic functional network pair combinations that characterised this network (listed in Table S1). For demographic and clinical measures that were significantly associated with connectivity measures, we tested if connectivity for the PTSD group continued to remain significantly associated with change in PTSD symptoms after controlling for these demographic and clinical measures.

***Results:***

For the whole PTSD group, average connectivity in the signature was found to be significantly associated with comorbidity (p=.037) and DASS measures of anxiety (p=.046). A post-hoc univariate analysis confirmed that when controlling for both these measures, symptom improvement was still significantly associated with the average connectivity within the connectomic signature (p<.001 for symptom improvement).

Age, gender, baseline symptom severity, DASS measures of depression and stress, SSRI use, trauma type and months since trauma were not found to be associated with any of the connectivity measures.

**Supplementary Table S1: Network-based statistical analysis identifying the relationship between baseline functional connectivity and improvement in CAPS scores for PSTD patients.** P-values for each link are also listed. The seed and target regions for the significant connections from this identified connectomic network were defined as belonging to the intrinsic functional brain networks based on the established parcellation template of the Gordon atlas rather than by anatomical boundaries (which means that an anatomical region could have membership for multiple intrinsic functional network due to multiple nodes representing the region). 25 unique network-pair combinations were identified and the individual connections comprising each network pair are listed.

| **Significant connections** | | | | |
| --- | --- | --- | --- | --- |
| **Region** | **Coordinates (x,y,z)** | **Region** | **Coordinates (x,y,z)** | **P-Value** |
| **CinguloOpercular - CinguloOpercular (4 links)** |  |  |  |  |
| L - Cingulate Gyrus (BA 32) | (-8.4, 14.6, 33.8) | L - Inferior Parietal Lobule (BA 40) | (-57.7, -40.6, 35.8) | .002 |
| L - Cingulate Gyrus (BA 32) | (-8.4, 14.6, 33.8) | L - Precentral Gyrus (BA 44) | (-48.6, 7.5, 11.1) | .001 |
| L - Inferior Parietal Lobule (BA 40) | (-57.7, -40.6, 35.8) | R -Cingulate Gyrus (BA 32) | (6, 21.8, 32.4) | .001 |
| L - Inferior Parietal Lobule (BA 40) | (-57.7, -40.6, 35.8) | R - Supramarginal Gyrus (BA 40) | (57.5, -40.3, 34.7) | .002 |
|  |  |  |  |  |
| **DMN - DMN (3 links)** |  |  |  |  |
| L - Superior Temporal Gyrus (BA 39) | (-47.2, -58, 30.8) | R - Superior Frontal Gyrus (BA 9) | (21.4, 42.8, 35.1) | .001 |
| L - Precuneus (BA 19) | (-39.3, -73.9, 38.3) | R - Superior Frontal Gyrus (BA 9) | (21.4, 42.8, 35.1) | .002 |
| L - Superior Temporal Gyrus (BA 39) | (-47.2, -58, 30.8) | R - Superior Frontal Gyrus (BA 6) | (11.9, 21.9, 59.9) | .002 |
|  |  |  |  |  |
| **Dorsal Attention -Dorsal Attention (3 links)** |  |  |  |  |
| L - Middle Frontal Gyrus (BA 6) | (-27.3, -6.8, 46.3) | L - Sub-Gyral (BA 6) | (-21.3, -0.2, 62.7) | .001 |
| L - Middle Frontal Gyrus (BA 6) | (-27.3, 1.9, 52.9) | L - Sub-Gyral (BA 6) | (-21.3, -0.2, 62.7) | .001 |
| L - Medial Frontal Gyrus (BA 6) | (-19.8, 6.4, 55.7) | L - Sub-Gyral (BA 6) | (-21.3, -0.2, 62.7) | .001 |
|  |  |  |  |  |
| **CinguloOpercular– Dorsal Attention (8 links)** |  |  |  |  |
| L - Anterior Cingulate (BA 32) | (-9, 25.3, 27.7) | L- Insula (BA 13) | (-41.6, 8.7, 22.2) | .001 |
| R - Cingulate Gyrus (BA 32) | (6, 21.8, 32.4) | L - Insula (BA 13) | (-41.6, 8.7, 22.2) | .002 |
| L - Anterior Cingulate (BA 32) | (-9, 25.3, 27.7) | R - Middle Frontal Gyrus (BA 10) | (36.8, 37.8, 13.1) | .001 |
| R - Cingulate Gyrus (BA 32) | (6, 21.8, 32.4) | R - Middle Frontal Gyrus (BA 10) | (36.8, 37.8, 13.1) | .001 |
| L - Cingulate Gyrus (BA 24) | (-9.4, -0.1, 42.9) | R - Angular Gyrus (BA 39) | (32.3, -63.6, 33.8) | .002 |
| R - Cingulate Gyrus (BA 24) | (8.6, 4.2, 40.1) | R - Angular Gyrus (BA 39) | (32.3, -63.6, 33.8) | >.001 |
| R - Cingulate Gyrus (BA 32) | (6, 21.8, 32.4) | R - Angular Gyrus (BA 39) | (32.3, -63.6, 33.8) | .001 |
| L - Cingulate Gyrus (BA 32) | (-8.4, 14.6, 33.8) | R - Middle Temporal Gyrus (BA 21) | (57, -53.8, -1.1) | .001 |
|  |  |  |  |  |
| **CinguloOpercular – Central Executive (13 links)** |  |  |  |  |
| L - Cingulate Gyrus (BA 32) | (-8.4, 14.6, 33.8) | R - Middle Temporal Gyrus (BA 21) | (59.7, -41, -10.9) | >.001 |
| L - Cingulate Gyrus (BA 32) | (-8.4, 14.6, 33.8) | R - Inferior Parietal Lobule (BA 40) | (41.5, -53.5, 44) | .001 |
| L - Cingulate Gyrus (BA 32) | (-8.4, 14.6, 33.8) | R - Middle Frontal Gyrus (BA 46) | (41.8, 29.1, 21.6) | .001 |
| L - Anterior Cingulate (BA 32) | (-9, 25.3, 27.7) | L - Middle Frontal Gyrus (BA 46) | (-40.2, 23.6, 23.3) | .002 |
| L - Anterior Cingulate (BA 32) | (-9, 25.3, 27.7) | R - Inferior Parietal Lobule (BA 40) | (47.9, -42.5, 41.5) | >.001 |
| L - Anterior Cingulate (BA 32) | (-9, 25.3, 27.7) | R - Middle Frontal Gyrus (BA 46) | (41.8, 29.1, 21.6) | >.001 |
| L – Claustrum | (-37.3, 8.9, -0.9) | R -Inferior Parietal Lobule (BA 40) | (41.5, -53.5, 44) | .002 |
| R - Cingulate Gyrus (BA 32) | (6, 21.8, 32.4) | L - Middle Frontal Gyrus (BA 46) | (-40.2, 23.6, 23.3) | .002 |
| R - Cingulate Gyrus (BA 32) | (6, 21.8, 32.4) | R - Inferior Parietal Lobule (BA 40) | (47.9, -42.5, 41.5) | .001 |
| R - Cingulate Gyrus (BA 32) | (6, 21.8, 32.4) | R - Inferior Parietal Lobule (BA 40) | (41.5, -53.5, 44) | >.001 |
| R - Cingulate Gyrus (BA 32) | (6, 21.8, 32.4) | R - Middle Frontal Gyrus (BA 46) | (41.8, 29.1, 21.6) | >.000 |
|  |  |  |  |  |
| **DMN – Dorsal Attention (1 link)** |  |  |  |  |
| R - Supramarginal Gyrus (BA 40) | (48.9, -53, 28.6) | L - Middle Frontal Gyrus (BA 6) | (-27.3, -6.8, 46.3) | .001 |
|  |  |  |  |  |
| **DMN – Central Executive (3 links)** |  |  |  |  |
| R - Middle Temporal Gyrus (BA 21) | (59.7, -41, -10.9) | L - Cingulate Gyrus (BA 24) | (-1.7, -17.7, 39.1) | .001 |
| R - Middle Temporal Gyrus (BA 21) | (59.7, -41, -10.9) | R - Cingulate Gyrus (BA 24) | (3, -19.6, 37.9) | .001 |
| R - Middle Temporal Gyrus (BA 21) | (59.7, -41, -10.9) | R - Middle Temporal Gyrus (BA 21) | (57.5, -7.4, -16.4) | .001 |
|  |  |  |  |  |
| **Dorsal Attention – Central Executive (3 links)** |  |  |  |  |
| L – Sub-Gyral (BA 6) | (-21.3, -0.2, 62.7) | R - Middle Temporal Gyrus (BA 21) | (59.7, -41, -10.9) | .002 |
| R – Middle Frontal Gyrus (BA 6) | (29.9, -7.8, 47.4) | R - Middle Frontal Gyrus (BA 10) | (42.8, 48.3, -5.1) | .002 |
| R – Inferior Parietal Lobule (BA 40) | (33.5, -48.2, 49.4) | R – Inferior Parietal Lobule (BA 40) | (35.7, -56.7, 45.2) | .001 |
|  |  |  |  |  |
| **CinguloOpercular – Auditory (5 links)** |  |  |  |  |
| R - Superior Frontal Gyrus (BA 6) | (6.7, 5, 55.9) | L – Insula (BA 13) | (-46.3, -41.4, 25.9) | .001 |
| R - Medial Frontal Gyrus (BA 32) | (8.8, 10.8, 45.9) | L – Insula (BA 13) | (-46.3, -41.4, 25.9) | .002 |
| R - Medial Frontal Gyrus (BA 32) | (8.8, 10.8, 45.9) | R – Insula (BA 13) | (59.2, -38.6, 14.6) | .001 |
| R - Middle Frontal Gyrus (BA 6) | (42.5, -2.3, 47.2) | L – Insula (BA 13) | (-46.3, -41.4, 25.9) | .002 |
| R - Middle Frontal Gyrus (BA 6) | (42.5, -2.3, 47.2) | R – Insula (BA 13) | (59.2, -38.6, 14.6) | .001 |
|  |  |  |  |  |
| **Dorsal Attention – Auditory (2 link)** |  |  |  |  |
| L - Medial Frontal Gyrus (BA 6) | (-19.8, 6.4, 55.7) | L - Superior Temporal Gyrus (BA 22) | (-59.6, -38.5, 16.5) | .001 |
| R - Inferior Parietal Lobule (BA 40) | (38.8, -42.6, 40.4) | R - Insula (BA 13) | (59.2, -38.6, 14.6) | .002 |
|  |  |  |  |  |
| **Central Executive – Auditory (5 links)** |  |  |  |  |
| R – Inferior Parietal Lobule (BA 40) | (47.9, -42.5, 41.5) | R – Insula (BA 13) | (59.2, -38.6, 14.6) | .001 |
| R – Inferior Parietal Lobule (BA 40) | (47.9, -42.5, 41.5) | R – Superior Temporal Gyrus (BA 42) | (60, -25.2, 10.2) | .001 |
| R - Inferior Parietal Lobule (BA 40) | (41.5, -53.5, 44) | L – Superior Temporal Gyrus (BA 22) | (-59.6, -38.5, 16.5) | .001 |
| R - Inferior Parietal Lobule (BA 40) | (41.5, -53.5, 44) | R – Insula (BA 13) | (59.2, -38.6, 14.6) | .001 |
| R - Inferior Parietal Lobule (BA 40) | (35.7, -56.7, 45.2) | R – Insula (BA 13) | (59.2, -38.6, 14.6) | .001 |
|  |  |  |  |  |
| **CinguloOpercular - Visual (7 links)** |  |  |  |  |
| L - Medial Frontal Gyrus (BA 6) | (-8, -8.7, 62.9) | R - Cuneus (BA 18) | (22, -84.6, 23.7) | .001 |
| R - Medial Frontal Gyrus (BA 6) | (8 ,-6.2, 63.7) | R - Cuneus (BA 18) | (22, -84.6, 23.7) | .001 |
| L - Precentral Gyrus (BA 6) | (-42.1, -4.5, 47.3) | R - Superior Occipital Gyrus (BA 19) | (35.4, -77.1, 21.1) | .001 |
| R - Medial Frontal Gyrus (BA 6) | (8, -6.2, 63.7) | R - Superior Occipital Gyrus (BA 19) | (35.4, -77.1, 21.1) | .002 |
| L - Cingulate Gyrus (BA 32) | (-8.4, 14.6, 33.8) | R - Superior Temporal Gyrus (BA 39) | (49, -54.5, 8.8) | .001 |
| L - Anterior Cingulate (BA 32) | (-9, 25.3, 27.7) | R - Superior Temporal Gyrus (BA 39) | (49, -54.5, 8.8) | .002 |
| L - Medial Frontal Gyrus (BA 6) | (-8, -8.7, 62.9) | R - Middle Occipital Gyrus (BA 18) | (13.8, -92.3, 14.7) | .001 |
|  |  |  |  |  |
| **Dorsal Attention – Visual (9 links)** |  |  |  |  |
| L - Middle Frontal Gyrus (BA 6) | (-27.3, -6.8, 46.3) | R - Cuneus (BA 18) | (22, -84.6, 23.7) | .001 |
| R - Middle Frontal Gyrus (BA 6) | (29.9, -7.8, 47.4) | R - Cuneus (BA 18) | (22, -84.6, 23.7) | >.001 |
| L - Middle Frontal Gyrus (BA 6) | (-27.3, -6.8, 46.3) | R - Precuneus (BA 7) | (17.6, -78.3, 34) | >.001 |
| L - Middle Frontal Gyrus (BA 6) | (-27.3, 1.9, 52.9) | R - Cuneus (BA 19) | (7.7, -85.6, 31.6) | .001 |
| L - Middle Frontal Gyrus (BA 6) | (-27.3, -6.8, 46.3) | R - Superior Occipital Gyrus (BA 19) | (35.4, -77.1, 21.1) | >.001 |
| L - Sub-Gyral (BA 6) | (-21.3, -0.2, 62.7) | R - Superior Occipital Gyrus (BA 19) | (35.4, -77.1, 21.1) | .002 |
| R - Middle Frontal Gyrus (BA 6) | (29.9, -7.8, 47.4) | R - Superior Occipital Gyrus (BA 19) | (35.4, -77.1, 21.1) | .001 |
| R - Middle Frontal Gyrus (BA 6) | (29.9, -7.8, 47.4) | R - Middle Occipital Gyrus (BA 19) | (31.7, -85.7, 2.4) | >.001 |
| L - Medial Frontal Gyrus (BA 6) | (-19.8, 6.4, 55.7) | R - Superior Temporal Gyrus (BA 39) | (49, -54.5, 8.8) | .002 |
|  |  |  |  |  |
| **CinguloOpercular - Subcortical (3 links)** |  |  |  |  |
| L - Anterior Cingulate (BA 32) | (-9, 25.3, 27.7) | L - Pallidum | (-19, 0, 0) | .001 |
| R - Cingulate Gyrus (BA 32) | (6, 21.8, 32.4) | L – Pallidum | (-19, 0, 0) | .002 |
| L - Anterior Cingulate (BA 32) | (-9, 25.3, 27.7) | R – Pallidum | (20, 0, 0) | >.001 |
|  |  |  |  |  |
| **DMN – Subcortical (11 links)** |  |  |  |  |
| R - Supramarginal Gyrus (BA 40) | (48.9, -53, 28.6) | L – Pallidum | (-19, 0, 0) | .001 |
| R - Angular Gyrus (BA 39) | (46.5, -67.3, 36.2) | L – Pallidum | (-19, 0, 0) | .001 |
| L - Anterior Cingulate (BA 32) | (-6, 44.9, 6.3) | R – Pallidum | (20, 0, 0) | .001 |
| R - Anterior Cingulate (BA 32) | (7.7, 44.1, 5.5) | R – Pallidum | (20, 0, 0) | .002 |
| R - Supramarginal Gyrus (BA 40) | (48.9, -53, 28.6) | R – Pallidum | (20, 0, 0) | >.001 |
| R - Angular Gyrus (BA 39) | (46.5, -67.3, 36.2) | R – Pallidum | (20, 0, 0) | >.001 |
| L - Anterior Cingulate (BA 32) | (-6, 44.9, 6.3) | L – Putamen | (-25, 4, 2) | .001 |
| R - Supramarginal Gyrus (BA 40) | (48.9, -53, 28.6) | L – Putamen | (-25, 4, 2) | >.001 |
| R - Angular Gyrus (BA 39) | (46.5, -67.3, 36.2) | L – Putamen | (-25, 4, 2) | >.001 |
| R - Supramarginal Gyrus (BA 40) | (48.9, -53, 28.6) | R - Putamen | (27, 5, 2) | >.001 |
| R - Angular Gyrus (BA 39) | (46.5, -67.3, 36.2) | R - Putamen | (27, 5, 2) | >.001 |
|  |  |  |  |  |
| **Central Executive - Subcortical (2 links)** |  |  |  |  |
| R – Middle Frontal Gyrus (BA 9) | (38.6, 18.8, 25.5) | R - Putamen | (27, 5, 2) | >.001 |
| R - Precentral Gyrus (BA 9) | (38.9, 9.6, 42.7) | R - Putamen | (27, 5, 2) | .002 |
|  |  |  |  |  |
| **Salience - Subcortical (6 links)** |  |  |  |  |
| L - Anterior Cingulate (BA 32) | (-10, 33.9, 21.5) | L - Pallidum | (-19, 0, 0) | >.001 |
| R - Anterior Cingulate (BA 32) | (8.4, 34.7, 22.6) | L - Pallidum | (-19, 0, 0) | .002 |
| L - Anterior Cingulate (BA 32) | (-10, 33.9, 21.5) | R – Pallidum | (20, 0, 0) | >.001 |
| L - Anterior Cingulate (BA 32) | (-10, 33.9, 21.5) | L – Putamen | (-25, 4, 2) | >.001 |
| R - Anterior Cingulate (BA 32) | (8.4, 34.7, 22.6) | L – Putamen | (-25, 4, 2) | .001 |
| L - Anterior Cingulate (BA 32) | (-10, 33.9, 21.5) | R - Putamen | (27, 5, 2) | .002 |
|  |  |  |  |  |
| **Somatomotor Hand – Visual (14 links)** |  |  |  |  |
| L - Medial Frontal Gyrus (BA 6) | (-5, -28.2, 60.4) | L - Middle Occipital Gyrus (BA 19) | (-31.3, -84.2, 9) | >.001 |
| L - Paracentral Lobule (BA 31) | (-5.4, -15.9, 48.8) | L - Middle Occipital Gyrus (BA 19) | (-31.3, -84.2, 9) | .001 |
| R - Medial Frontal Gyrus (BA 6) | (4.8, -27.1, 64.8) | L - Middle Occipital Gyrus (BA 19) | (-31.3, -84.2, 9) | .001 |
| R - Medial Frontal Gyrus (BA 6) | (5.1, -17.1, 51.6) | L - Middle Occipital Gyrus (BA 19) | (-31.3, -84.2, 9) | .002 |
| L - Paracentral Lobule (BA 31) | (-5.4, -15.9, 48.8) | L - Inferior Occipital Gyrus (BA 19) | (-41.2, -72.1, -5.9) | .002 |
| R - Postcentral Gyrus (BA 3) | (11.9, -40.7, 67) | L - Inferior Occipital Gyrus (BA 19) | (-41.2, -72.1, -5.9) | .001 |
| R - Medial Frontal Gyrus (BA 6) | (5.1, -17.1, 51.6) | L - Inferior Occipital Gyrus (BA 19) | (-41.2, -72.1, -5.9) | .001 |
| R - Medial Frontal Gyrus (BA 6) | (12.4, -28.3, 69.6) | L - Inferior Occipital Gyrus (BA 19) | (-41.2, -72.1, -5.9) | .001 |
| L - Paracentral Lobule (BA 31) | (-5.4, -15.9, 48.8) | R - Cuneus (BA 18) | (22, -84.6, 23.7) | .001 |
| R - Cingulate Gyrus (BA 24) | (6.8, -8.1, 50.9) | R - Cuneus (BA 18) | (22, -84.6, 23.7) | .001 |
| L - Middle Frontal Gyrus (BA 6) | (-17.2, -8.6, 67.9) | R - Precuneus (BA 7) | (17.6, -78.3, 34) | .001 |
| R - Medial Frontal Gyrus (BA 6) | (5.1, -17.1, 51.6) | R - Middle Occipital Gyrus (BA 37) | (43.8, -67.2, 2) | .001 |
| L - Paracentral Lobule (BA 31) | (-5.4, -15.9, 48.8) | R - Middle Occipital Gyrus (BA 18) | (13.8, -92.3, 14.7) | .001 |
| R - Medial Frontal Gyrus (BA 6) | (5.1, -17.1, 51.6) | R - Middle Occipital Gyrus (BA 18) | (13.8, -92.3, 14.7) | .001 |
|  |  |  |  |  |
| **Central Executive – Salience (2 links)** |  |  |  |  |
| R - Middle Frontal Gyrus (BA 46) | (41.8, 29.1, 21.6) | L - Anterior Cingulate (BA 32) | (-10, 33.9, 21.5) | >.001 |
| R - Middle Frontal Gyrus (BA 46) | (41.8, 29.1, 21.6) | R - Anterior Cingulate (BA 32) | (8.4, 34.7, 22.6) | .002 |
|  |  |  |  |  |
| **DMN – Salience (1 links)** |  |  |  |  |
| L - Superior Temporal Gyrus (BA 39) | (-47.2, -58, 30.8) | R - Anterior Cingulate (BA 32) | (8.4, 34.7, 22.6) | .001 |
|  |  |  |  |  |
| **Salience – Salience (1 links)** |  |  |  |  |
| L - Anterior Cingulate (BA 32) | (-10, 33.9, 21.5) | L - Inferior Frontal Gyrus (BA 47) | (-32.5, 17.2, -7.8) | .001 |
|  |  |  |  |  |
| **CinguloOpercular – Somatomotor Hand (1 link)** |  |  |  |  |
| R - Superior Frontal Gyrus (BA 6) | (6.7, 5, 55.9) | R - Superior Frontal Gyrus (BA 6)-R | (17, -16.9, 70.9) | .001 |
|  |  |  |  |  |
| **Dorsal Attention – Somatomotor Hand (2 links)** |  |  |  |  |
| L - Middle Frontal Gyrus (BA 6) | (-27.3, -6.8, 46.30 | R - Inferior Frontal Gyrus (BA 11) | (10.9, 39.1, -19.7) | .001 |
| R - Middle Frontal Gyrus (BA 6) | (29.9, -7.8, 47.4) | R - Inferior Frontal Gyrus (BA 11) | (10.9, 39.1, -19.7) | .001 |
|  |  |  |  |  |
| **Central Executive – Ventral Attention (1 link)** |  |  |  |  |
| R - Inferior Parietal Lobule (BA 40) | (47.9, -42.5, 41.5) | R- Superior Temporal Gyrus (BA 41) | (47.4, -39.6, 13.2) | .002 |
|  |  |  |  |  |
| **CinguloOpercular – Ventral Attention (1 link)** |  |  |  |  |
| L - Anterior Cingulate (BA 32) | (-9, 25.3, 27.7) | R - Superior Temporal Gyrus (BA 22) | (57.5, -45.3, 9) | .002 |

**S3: Predictive models using the identified connectomic signature in classifying responders from non-responders prior to treatment**

We examined the predictive performance of the identified connectomic signature in classifying PTSD responders from PTSD non-responders prior to treatment using cross-validation analyses. To illustrate how the identified functional markers could prove helpful in a treatment decision and to inform future studies, we first evaluated the predictive performance of a demographic and clinical measures model, and whether its performance was improved by using connectivity estimates for our identified connectomic signature. First, we noted the demographic and clinical features that distinguished responders from non-responders, and the significant variables were included in the first block of a binary logistic regression. In the same model, as block 2, the 25 averaged network connectivity measures (in Table S3) identified in primary analyses were entered in a forward stepwise (Wald) method, to determine which variables contributed significantly to the model, and whether those variables improved the model’s prediction of treatment response above and beyond clinical variables.

After identifying the variables that significantly contributed to improved prediction of treatment response, we ran three cross-validation models: 1) using only the demographic and clinical features that distinguished response were input in this clinical-only model; 2) using the identified connectivity values for the individual network pairs that comprised of the connectomic signature from the above forward stepwise regression; and 3) using the overall average connectivity measures for the connectomic signature. This was mainly done to identify the most predictive features within the connectomic signature. The dataset was randomly split into an approximately 60% training dataset and 40% testing dataset. The training dataset was then bootstrapped two hundred times, and binary logistic regressions were performed on each bootstrapped dataset. Model coefficients from each predictor were extracted and averaged across all results. These averaged coefficients were then used in a binary logistic regression model on the test data to determine the model’s cross-validated accuracy statistics for classifying PTSD treatment responder's vs non-responders. Accuracy statistics (sensitivity, specificity, positive predictive values, and negative predictive values) are reported for the training models as well as those that were derived from cross-validation.

***Results:***

Only DASS anxiety scores differed between treatment responders and non-responders groups (PTSD-R < PTSD-NR) and was used as the sole feature for the block 1 clinical variables only model, which did not significantly predict treatment response (p=0.054). There were no significant differences for other variables (gender, age, SSRI use, DASS depression or stress scores, trauma type, and months since trauma). For block 2, the neural measures that were retained at the end of the forward stepwise regression were: connectivity between the central executive/fronto parietal (FPN) and CingularOpercular (CO) network and that between the CinguloOpercular and Somotomotor (SMH) network regions. The combined models using the whole sample were significantly improved as compared to the clinical only block 1 model (p<0.001). The split-sample training and cross-validated model accuracies are reported in Supplementary Table S2.

**Supplementary Table S2: Predictive Models using demographic/clinical measures alone and combined models using connectivity measures for the identified connectomic signatures. Both combined models improved predictive accuracies as compared to the clinical only model to classify prospective responders from non-responders.**

|  | **Variable** | **Coefficient β values** | **% accuracy**  **(95% CI)** | **% specificity** | **%**  **sensitivity** | **Positive/Negative Predictive Value** |
| --- | --- | --- | --- | --- | --- | --- |
| ***Clinical only model*** | | | | | |  |
| *Training model* |  |  | 59.1%  (36-79%) | 80.0% | 14.3% | 25.0%/66.7% |
| *Cross-validated model* |  |  | 64.3%  (35-87%) | 80.0% | 25.0% | 33.3%/72.7% |
|  | DASS Anxiety | -0.28 |  |  |  |  |
| ***Combined Model 1*** | | | | | |  |
| *Training model* |  |  | 86.4%  (65-97%) | 93.3% | 71.4% | 83.3%/87.5% |
| *Cross-validated model* |  |  | 78.6%  (49-95%) | 90.0% | 50.0% | 66.7%/81.8% |
|  | DASS Anxiety | -6.59 |  |  |  |  |
|  | FPN-CO | -330.62 |  |  |  |  |
|  | CO – SMH | -34.05 |  |  |  |  |
| ***Combined Model 2*** | | | | | |  |
| *Training model* |  |  | 68.2%  (45-86%) | 80.0% | 42.8% | 50.0%/75.0% |
| *Cross-validated model* |  |  | 71.4%  (42-92%) | 60.0% | 100.0% | 50.0%/100.0% |
|  | DASS Anxiety | -3.15 |  |  |  |  |
|  | Overall average connectivity | -124.34 |  |  |  |  |

**S4: Network-Based Statistical analyses comparing whole PTSD group with Controls**

Whole brain connectivity comparisons between the entire PTSD group and controls (matched for age and gender) at baseline were conducted using NBS. A two-sample t-test at each edge independently to test for significant differences in the value of connectivity between the two groups was first performed at p<0.001 to identify components or sub-networks and then the statistical significance of the size of each observed component was then evaluated with respect to an empirical null distribution at a p<0.05 corrected for multiple comparisons. This analysis did not identify any significant differences in functional connectivity between the PTSD and control groups.

**S5: Replication of main connectome analyses using the AAL parcellation scheme**

The choice of a parcellation scheme can impact the results of a network analysis (9-11). To ensure that our findings are robust to the choice of a particular parcellation scheme, we also tested our findings using the AAL atlas which is an anatomical based parcellation and different from the Gordon atlas which is parcellated based on areas that have functional coherent resting state functional connectivity patterns. As done for the main analysis, resting fMRI time series were extracted for each of the AAL regions and inter-regional correlations were performed to obtain a 90 x 90 inter-regional connectivity matrix for every individual.

We performed two analyses: 1) First, we compared change in PTSD symptoms for the entire PTSD group using the whole 90x90 functional connectivity matrices in NBS. 2) Secondly, we mapped each of the AAL regions on to each of the 343 functional parcellation regions and identified all the links from the 343x343 connectome matrix which corresponded to those that were significant in the AAL based NBS analysis in 1. We then used multivariate ANOVA analyses to examine which of these links were significantly associated with treatment response. This approach serves to directly test the replicability of results across parcellation schemes and also labels the specific sub-regions of the AAL regions into the intrinsic functional networks.

***Results:*** 1) For the first NBS analysis using the AAL parcellation, a connectomic signature comprising of 12 edges connecting 9 nodes was significantly associated with treatment response for the PTSD group (corrected p<0.05). As observed in the main analysis, lower baseline functional connectivity was associated with better symptom improvement.

2) For the second analysis that mapped each of AAL regions on to the functional parcellation regions, a total 39 links were identified which corresponded to those in the identified AAL based network. Of these, connectivity in 22 links were associated with significant changes in CAPS score following TF-CBT, with 10/12 edges from the AAL based connectome analysis found to replicate (supplementary table S3). These data suggest that our findings were robust to our choice of parcellation scheme. However, it is important to note that many of the significant network connections from our analysis using the AAL parcellation mapped onto subcortical AAL regions that were included in our main analysis rather than Gordon defined parcels. Furthermore, a small sample size in conjunction with a stringent primary component-forming threshold (p<.001) for our NBS analyses using the AAL atlas, may account for why significant network connections (i.e. connections from the right angular gyrus to the left angular gyrus) did not replicate. There were however trend level significance for the remaining 2 links.

**Supplementary Table S3: Pretreatment network identified to be significant with improvement in symptoms using network-based statistical analysis based on parcellations derived from the AAL atlas.** Intrinsic brain networks associated with each connection identified from the functional parcellation are listed along with p-values. *Abbreviations*: AAL, automated, anatomical labeling; CO, cingulo-opercular; DAN, dorsal attention; DMN, default mode network; FPN, fronto-parietal; L, Left; R, Right; SMH, somatomotor hand.

|  |  |  |
| --- | --- | --- |
| **Significant Network Connections (AAL Regions)** |  | **p-value** |
|  | **Associated intrinsic brain networks.** |  |
|  |  |  |
|  |  |  |
| *Angular (R) to Angular (L) | DMN to DMN & FPN to DMN | 0.074 |
| *Superior Frontal (R) to Angular (L) | FPN to DMN, DMN to DMN, DAN to DMN & SMH to DMN | 0.097 |
| Anterior Cingulum (R) to Pallidum (R) | DMN to Subcortical, Salience to Subcortical | 0.004 |
| Anterior Cingulum (L) to Pallidum (R) | CO to Subcortical, DMN to Subcortical & Salience to Subcortical | 0.001 |
| Putamen (L) to Angular (R) | Subcortical to FPN & Subcortical to DMN | <0.001 |
| Putamen (R) to Angular (R) | Subcortical to FPN, Subcortical to DMN | <0.001 |
| Pallidum (L) to Angular (R) | Subcortical to FPN, Subcortical to DMN | 0.007 |
| Pallidum (R) to Angular (R) | Subcortical to FPN, Subcortical to DMN | <0.001 |
| Anterior Cingulum (L) to Putamen (L) | CO to Subcortical, DMN to Subcortical, Salience to Subcortical | 0.001 |
| Anterior Cingulum (R) to Putamen (L) | DMN to Subcortical, Salience to Subcortical | 0.003 |
| Anterior Cingulum (R) to Pallidum (L) | DMN to Subcortical, Salience to Subcortical | 0.007 |
| Anterior Cingulum (R) to Angular (L) | DMN to DMN, & Salience to DMN | 0.004 |
|  |  |  |
| *Network links that did not replicate with the functional parcellation | |  |

**S6: Testing effects of current antidepressant medication use on findings**

To examine if the current associations between pretreatment functional connectivity and PTSD symptom change were not confounded by use of antidepressant medications, we re-tested the significant connectivity correlations including only the 26 patients not on any current antidepressants at time of TF-CBT treatment (Table S4).

For the average connectivity in the identified signature and each individual intra & inter network connection of the identified signature, associations between symptom change and pre-treatment neural connectivity measures were not confounded by antidepressant use.

**Supplementary Table S4: Pretreatment functional connectivity associations with improvement in PTSD CAPS scores in non-medicated PTSD patient group.**

| **Significant Network Connections** | **Pearson**  **Correlation** | **p-value** |
| --- | --- | --- |
| Average Connectivity for the Signature | -0.840 | <0.001 |
| CinguloOpercular - CinguloOpercular | -0.667 | <0.001 |
| DMN - DMN | -0.620 | 0.001 |
| Dorsal Attention -Dorsal Attention | -0.645 | <0.001 |
| CinguloOpercular– Dorsal Attention | -0.732 | <0.001 |
| CinguloOpercular – Central Executive | -0.810 | <0.001 |
| DMN – Dorsal Attention | -0.442 | 0.027 |
| DMN – Central Executive | -0.582 | 0.002 |
| Dorsal Attention – Central Executive | -0.657 | <0.001 |
| CinguloOpercular – Auditory | -0.648 | <0.001 |
| Dorsal Attention – Auditory | -0.705 | <0.001 |
| Central Executive – Auditory | -0.593 | 0.002 |
| CinguloOpercular – Visual | -0.684 | <0.001 |
| Dorsal Attention – Visual | -0.693 | <0.001 |
| CinguloOpercular – Subcortical | -0.541 | 0.005 |
| DMN – Subcortical | -0.723 | <0.001 |
| Central Executive – Subcortical | -0.496 | 0.012 |
| Salience – Subcortical | -0.553 | 0.004 |
| Somatomotor Hand – Visual | -0.774 | <0.001 |
| Central Executive – Salience | -0.565 | 0.003 |
| DMN – Salience | -0.553 | 0.004 |
| Salience – Salience | -0.532 | 0.006 |
| CinguloOpercular – Somatomotor Hand | -0.554 | 0.004 |
| Dorsal Attention – Somatomotor Hand | -0.627 | 0.001 |
| Central Executive – Ventral Attention | -0.574 | 0.003 |
| CinguloOpercular – Ventral Attention | -0.465 | 0.019 |

**References:**

1. Korgaonkar MS, Grieve SM, Etkin A, Koslow SH, Williams LM. Using standardized fMRI protocols to identify patterns of prefrontal circuit dysregulation that are common and specific to cognitive and emotional tasks in major depressive disorder: first wave results from the iSPOT-D study. Neuropsychopharmacology : official publication of the American College of Neuropsychopharmacology. 2013;38(5):863-71.

2. Bryant RB, Erlinger M, Felmingham K, Klimova A, Williams LM, Malhi G, et al. Reappraisal-Related Neural Predictors of Treatment Response to Cognitive Behavior Therapy for Posttraumatic Stress Disorder. Psychological Medicine (in press)

3. Power JD, Barnes KA, Snyder AZ, Schlaggar BL, Petersen SE. Spurious but systematic correlations in functional connectivity MRI networks arise from subject motion. NeuroImage. 2012;59(3):2142-54.

4. Power JD, Mitra A, Laumann TO, Snyder AZ, Schlaggar BL, Petersen SE. Methods to detect, characterize, and remove motion artifact in resting state fMRI. NeuroImage. 2014;84:320-41.

5. Siegel JS, Power JD, Dubis JW, Vogel AC, Church JA, Schlaggar BL, et al. Statistical improvements in functional magnetic resonance imaging analyses produced by censoring high-motion data points. Human brain mapping. 2014;35(5):1981-96.

6. Andersson JL, Jenkinson M, Smith SM. Non-linear optimisation. FMRIB technical report tr07ja1.

7. Friston KJ, Williams S, Howard R, Frackowiak RS, Turner R. Movement-related effects in fMRI time-series. Magnetic resonance in medicine. 1996;35(3):346-55.

8. Korgaonkar MS, Ram K, Williams LM, Gatt JM, Grieve SM. Establishing the resting state default mode network derived from functional magnetic resonance imaging tasks as an endophenotype: A twins study. Human brain mapping. 2014;35(8):3893-902.

9. Fornito A, Zalesky A, Bullmore ET. Network scaling effects in graph analytic studies of human resting-state FMRI data. Frontiers in systems neuroscience. 2010;4:22.

10. Wang J, Wang L, Zang Y, Yang H, Tang H, Gong Q, et al. Parcellation-dependent small-world brain functional networks: a resting-state fMRI study. Human brain mapping. 2009;30(5):1511-23.

11. Zalesky A, Fornito A, Harding IH, Cocchi L, Yucel M, Pantelis C, et al. Whole-brain anatomical networks: does the choice of nodes matter? NeuroImage. 2010;50(3):970-83.
